# Early versus delayed impella unloading during venoarterial ECMO for cardiogenic shock: a propensity-matched analysis

**DOI:** 10.1007/s10047-026-01606-2

**Published:** 2026-11-13

**Authors:** Aabha Divya, Niti Dalal

**Affiliations:** 1 Division of Cardiothoracic Surgery, Tulane University School of Medicine, 1430 Tulane Ave, New Orleans, LA 70112 USA; 2 Department of Anesthesiology and Perioperative Medicine, Tulane University School of Medicine, Murphy Building, LA 70112 New Orleans, USA

**Keywords:** Cardiogenic shock, VA-ECMO, Impella, Left ventricular unloading, Real-world data

## Abstract

Venoarterial extracorporeal membrane oxygenation (VA-ECMO) restores perfusion in cardiogenic shock but increases left-ventricular afterload. Impella unloading (ECPELLA) is increasingly used, but optimal timing remains uncertain. We retrospectively analyzed adults with cardiogenic shock treated with VA-ECMO and Impella from 2016 to 2025. Early ECPELLA was Impella placement from the calendar day before through the day after VA-ECMO initiation; delayed ECPELLA was placement 2–7 days after VA-ECMO initiation. One-to-one propensity-score matching balanced 35 covariates. The primary outcome was 30-day all-cause mortality. Sensitivity analyses included acute kidney injury (AKI) restriction, VA-ECMO-indexed follow-up, and a 7-day landmark analysis. Matching yielded 282 per group. Mortality was similar at 30 days (48.9% vs. 49.6%; risk ratio [RR] 0.99, 95% CI 0.83–1.17), 90 days (RR 1.01, 95% CI 0.87–1.17), and 1 year (RR 1.03, 95% CI 0.89–1.18). Early ECPELLA was associated with more renal-replacement therapy (RRT) at 30 days (37.4% vs. 21.7%; RR 1.73, 95% CI 1.29–2.31), but not when follow-up began at VA-ECMO initiation in AKI-restricted cohorts (RR 1.00, 95% CI 0.81–1.22; adjusted hazard ratio 1.02, 95% CI 0.82–1.26). Major bleeding, sepsis, and limb ischemia were similar. Durable LVAD implantation was less frequent with early ECPELLA at 1 year (RR 0.57, 95% CI 0.34–0.95), but not in sensitivity analyses. Early ECPELLA was not associated with improved survival compared with delayed ECPELLA. The RRT association was not reproduced with aligned follow-up. Residual confounding and immortal-time bias limit causal inference and warrant randomized trials.

**Graphical abstract:**

**Figure:**
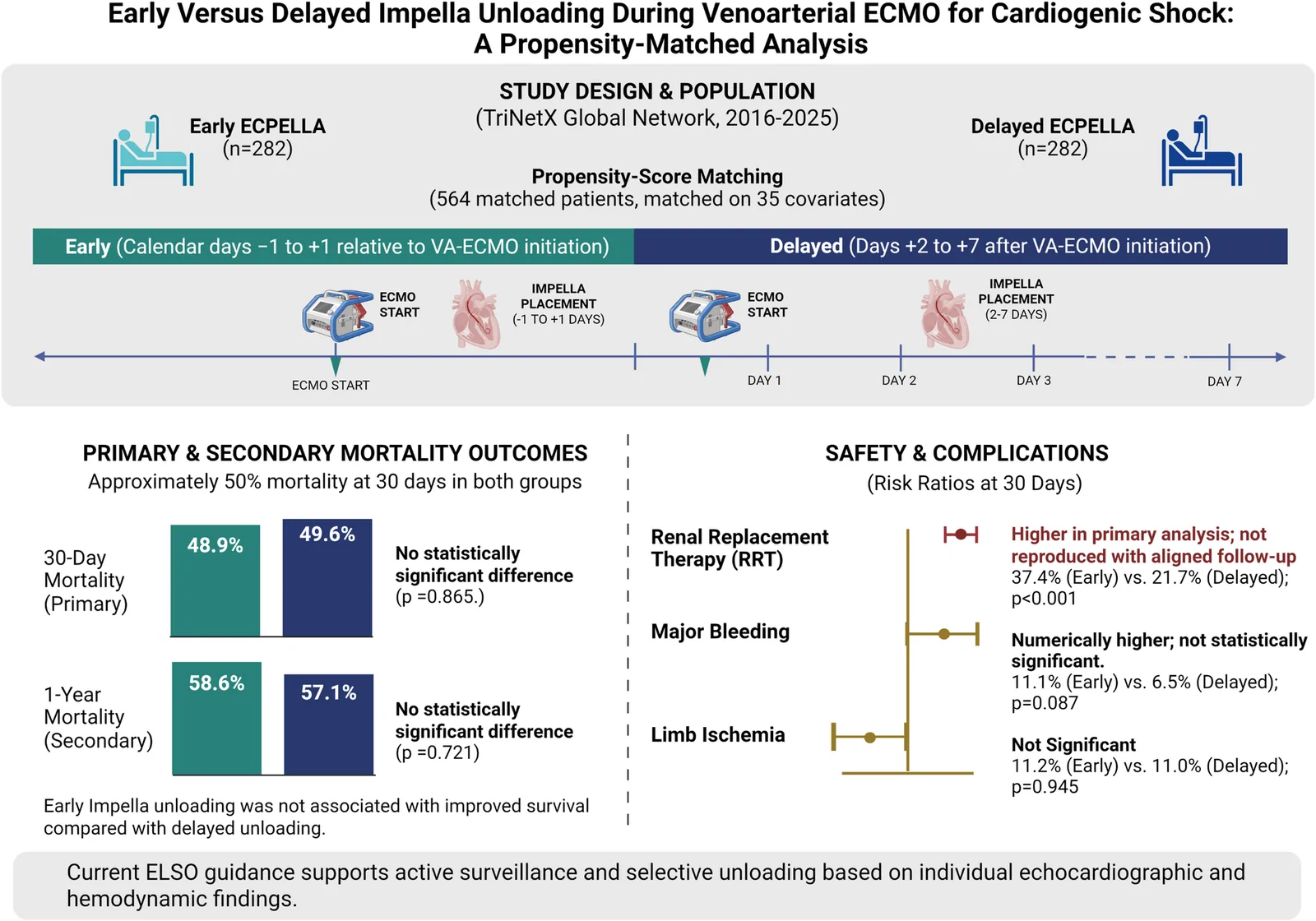

**Supplementary Information:**

The online version contains supplementary material available at https://doi.org/10.1007/s10047-026-01606-2.

## Introduction

Cardiogenic shock is the leading cause of in-hospital death after acute myocardial infarction and remains associated with a mortality of 40–50% despite advances in revascularization and supportive care [1, 2]. The 2022 ACC/AHA/HFSA guidelines and the 2021 ESC guidelines recognize short-term mechanical circulatory support (MCS) as a Class IIa option for patients with refractory shock, while emphasizing that evidence to guide device selection and timing remains limited [3, 4]. The Society for Cardiovascular Angiography and Interventions shock classification facilitates risk stratification and comparison across studies, with progressively higher mortality across advanced stages [5–8]. However, there is limited evidence regarding device selection, patient selection, and timing.

Venoarterial extracorporeal membrane oxygenation (VA-ECMO) provides rapid biventricular and respiratory support but may increase left ventricular (LV) afterload via retrograde aortic flow, and may worsen LV distension, pulmonary congestion, increase myocardial oxygen demand, and intracardiac stasis [9]. Routine early VA-ECMO did not improve survival and increased bleeding and vascular complications in the ECLS-SHOCK trial [1, 10]. By contrast, the DanGer Shock trial demonstrated a survival benefit with Impella CP in selected patients with ST-segment–elevation myocardial infarction complicated by cardiogenic shock [2, 11]. These findings have intensified interest in combining VA-ECMO for systemic perfusion with Impella-mediated LV unloading, commonly termed ECPELLA [12–14].

Observational data suggest that LV unloading during VA-ECMO is associated with lower mortality but with higher rates of hemolysis, renal-replacement therapy, and vascular complications [13, 14]. However, evidence regarding timing remains inconsistent. Schrage et al. reported that the survival association was strongest when Impella unloading was initiated before or shortly after VA-ECMO cannulation, whereas delayed unloading was not associated with a similar benefit [15]. However, these analyses were observational and did not provide a definitive direct comparison of planned early versus rescue unloading. Evidence regarding timing remains mixed. Grandin et al. found no significant mortality difference between upfront and delayed unloading, whereas Schrage et al. reported that unloading before or shortly after VA-ECMO initiation was associated with better survival [14, 15]. The randomized EARLY-UNLOAD trial, which evaluated transseptal left-atrial decompression rather than Impella, also found no reduction in 30-day mortality with routine early unloading [16]. The UNLOAD-ECMO randomized trial was initiated to compare Impella-mediated LV unloading in addition to VA-ECMO with VA-ECMO alone, although randomized results have not yet been published [17].

Given continued uncertainty and substantial variation in clinical practice, we used a large federated electronic health record network to compare early and delayed Impella unloading among adults receiving VA-ECMO for cardiogenic shock. We hypothesized that early ECPELLA would be associated with lower mortality but potentially greater device-related complications.

## Methods

### Data source

We performed a retrospective cohort study using the TriNetX Global Collaborative Network, a federated, de-identified electronic health-record platform that, at the time of analysis, included 173 healthcare organizations across multiple countries [18]. TriNetX harmonizes diagnoses (ICD-10-CM), procedures (CPT, ICD-10-PCS), medications, laboratory values, and demographics, and supports built-in propensity-score matching and survival analytics. Because the data are de-identified and aggregated at the network level, the analysis was exempt from institutional review board oversight under applicable regulations. TriNetX-based real-world evidence has been used to inform regulatory and comparative-effectiveness research [19].

### Study population and exposure definition

Eligible patients were adults aged 18 years or older with a diagnosis of cardiogenic shock (ICD-10-CM R57.0) who underwent VA-ECMO (CPT 33947 or ICD-10-PCS 5A1522G) between 1 January 2016 and 31 December 2025. Impella support was identified using the percutaneous left-heart ventricular-assist-device insertion code (CPT 33990). Because TriNetX records procedure dates without time stamps, exposure windows were defined in calendar days. Patients were classified as receiving early ECPELLA when Impella was placed on the calendar day before, the day of, or the calendar day after VA-ECMO initiation, and delayed ECPELLA when Impella was placed 2–7 days after VA-ECMO initiation. The early window was designed to capture Impella placement immediately before, concurrent with, or shortly after VA-ECMO initiation; hour-level definitions, such as placement within 2 h of cannulation used by Schrage et al. [15], could not be applied reliably. The delayed window was designed to capture subsequent or rescue unloading during the acute VA-ECMO course; its 7-day ceiling limited immortal-time bias and excluded later device escalation. In the primary analysis, the index date was the date on which all cohort criteria were first met: the later of VA-ECMO initiation and Impella insertion for early ECPELLA (at most 1 day after VA-ECMO initiation), and the date of Impella insertion (2–7 days after VA-ECMO initiation) for delayed ECPELLA.

### Outcomes

The primary outcome was 30-day all-cause mortality. Pre-specified secondary outcomes were 90-day and 1-year all-cause mortality, renal-replacement therapy (any of CPT 90935, 90937, 90945, 90947 or ICD-10-PCS 5A1D70Z), major bleeding (a composite of ICD-10-CM K92.2, I60, I61, I62 or R58), durable LVAD implantation (CPT 33979), sepsis (ICD-10-CM A41.9), and limb ischemia (ICD-10-CM I74.3, embolism and thrombosis of arteries of the lower extremities, or I99.8, other disorder of the circulatory system). Outcomes were assessed within follow-up windows beginning on the index date and extending to 30, 90, and 365 days. Patients with the outcome of interest before the start of the relevant window were excluded from that outcome-specific analysis to identify patients who were at risk.

### Propensity score matching

One-to-one nearest-neighbor propensity-score matching was performed using logistic regression with a caliper of 0.1 pooled standard deviations of the propensity score. Thirty-five pre-specified covariates, ascertained from records before the index date, were balanced, spanning demographics (age, sex, race,, and ethnicity, which included unknown categories), cardiovascular comorbidities (essential hypertension, diabetes mellitus, obesity, tobacco use, atherosclerotic coronary artery disease, acute myocardial infarction, heart failure with its subtypes, ischemic and dilated cardiomyopathy, end-stage heart failure, acute pulmonary oedema, cardiac arrest), renal disease (chronic kidney disease and acute kidney failure), and respiratory support (continuous positive-airway pressure of varying duration, mechanical ventilation of varying duration, endotracheal intubation and cardiopulmonary resuscitation). Balance was assessed using standardized mean differences (SMD); values < 0.1 were considered indicative of adequate balance. The same matched cohorts were used for the 30-day, 90-day, and 1-year analyses.

### Statistical analysis

Outcome analyses were performed on the matched cohorts. Categorical outcomes were summarized as risk, risk difference, risk ratio (RR), and odds ratio with 95% confidence intervals (CI). Time-to-event outcomes were summarized using Kaplan–Meier survival functions, log-rank tests, and Cox proportional hazards models, with assessment of the proportional hazards assumption and when the assumption was violated (*p* < 0.05), the RR was considered the primary estimate. Two-sided *p*-values < 0.05 were considered statistically significant. Because multiple outcomes were tested across three windows, the secondary results should be viewed as hypothesis-generating rather than confirmatory.

### Sensitivity analyses

We performed four sensitivity analyses. First, we compared outcomes in the unmatched cohorts to assess the effect of matching. Second, to address residual imbalance in acute kidney failure, we restricted both cohorts to patients with AKI (ICD-10-CM N17) documented on or before the day of VA-ECMO initiation (in the delayed cohort, on or before the first date on which cardiogenic shock, VA-ECMO, and AKI were all recorded) and repeated propensity-score matching. Third, to align the start of follow-up, we repeated the AKI-restricted analysis with the index date set to VA-ECMO initiation in both groups, using propensity-score matching and, separately, a multivariable Cox proportional hazards model for 30-day renal-replacement therapy adjusted for sex, AKI, and heart failure. Fourth, because classification as delayed ECPELLA required survival until Impella insertion, we performed a landmark analysis restricted to patients alive 7 days after VA-ECMO initiation; after repeat propensity-score matching, outcomes were assessed from day 8 through day 90 after the index date, defined as in the primary analysis (at most 1 day after VA-ECMO initiation for early ECPELLA; Impella insertion for delayed ECPELLA). The second through fourth analyses were performed after a subsequent network update (185–186 HCOs).

## Results

### Study cohorts and baseline characteristics

**Fig. 1:**
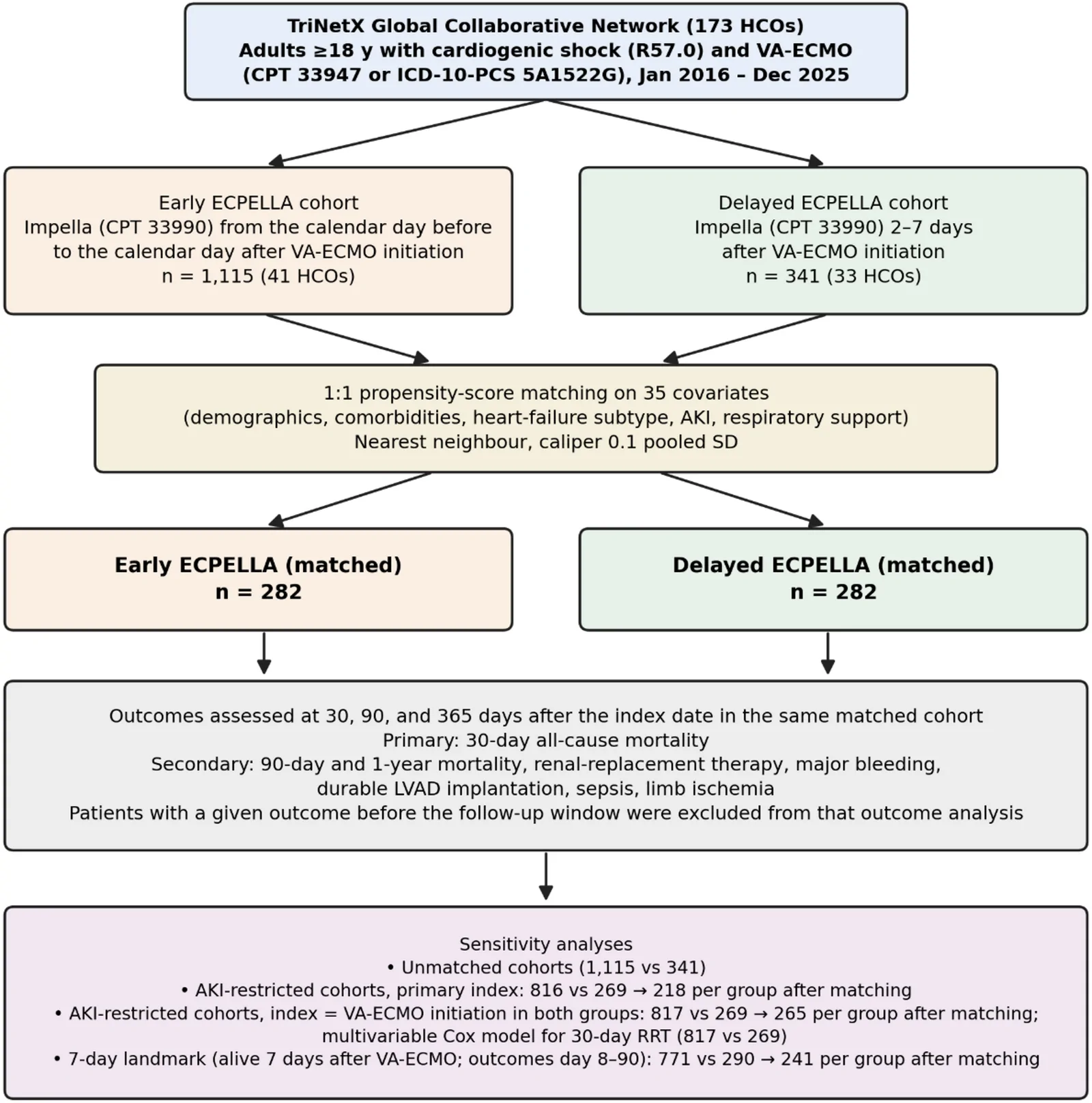
Study flow diagram. Patients with cardiogenic shock and venoarterial ECMO between January 2016 and December 2025 were classified as early ECPELLA (Impella from the calendar day before to the calendar day after VA-ECMO initiation) or delayed ECPELLA (Impella 2–7 days after VA-ECMO initiation). One-to-one propensity-score matching was performed on 35 covariates, and the same matched cohorts were used for outcomes at 30, 90, and 365 days. Sensitivity analyses are listed at the bottom. AKI, acute kidney injury; HCO, healthcare organization; LVAD, left ventricular assist device; RRT, renal-replacement therapy; SD, standard deviation; VA-ECMO, venoarterial extracorporeal membrane oxygenation

The study flow is shown in Fig. 1. Before matching, 1,115 patients received early ECPELLA and 341 received delayed ECPELLA. After 1:1 propensity-score matching, 282 patients remained in each group; the same matched cohorts were used for the 30-day, 90-day, and 1-year analyses. Baseline characteristics after matching are summarized in Table 1, and characteristics before and after matching in Supplementary Table S1.

**Table 1:** Baseline characteristics of the matched cohorts (30-day analysis, *N* = 282 per group)

Characteristic	Early ECPELLA (*n* = 282)	Delayed ECPELLA (*n* = 282)	SMD
*Demographics*			
Age at index, years, mean ± SD	57.7 ± 13.1	57.3 ± 13.3	0.031
Female	79 (28.0%)	76 (27.0%)	0.024
Black or African American	36 (12.8%)	32 (11.3%)	0.044
Male	203 (72.0%)	206 (73.0%)	0.024
White	196 (69.5%)	194 (68.8%)	0.015
American Indian or Alaska Native	≤ 10	≤ 10	< 0.001
Unknown race	28 (9.9%)	29 (10.3%)	0.012
Native Hawaiian or Other Pacific Islander	≤ 10	≤ 10	< 0.001
Unknown ethnicity	26 (9.2%)	30 (10.6%)	0.047
Hispanic or Latino	15 (5.3%)	17 (6.0%)	0.031
Not Hispanic or Latino	241 (85.5%)	235 (83.3%)	0.059
Other race	12 (4.3%)	15 (5.3%)	0.050
Asian	≤ 10	≤ 10	< 0.001
*Comorbidities and acute diagnoses*			
Essential hypertension (I10)	160 (56.7%)	153 (54.3%)	0.050
Diabetes mellitus (E08–E13)	128 (45.4%)	122 (43.3%)	0.043
Chronic kidney disease (N18)	101 (35.8%)	97 (34.4%)	0.030
Atherosclerotic coronary artery disease (I25.10)	212 (75.2%)	198 (70.2%)	**0.112**
Heart failure, any (I50)	263 (93.3%)	255 (90.4%)	**0.104**
Overweight and obesity (E66)	132 (46.8%)	123 (43.6%)	0.064
Tobacco use (Z72.0)	38 (13.5%)	36 (12.8%)	0.021
Cardiac arrest (I46)	127 (45.0%)	117 (41.5%)	0.072
Acute myocardial infarction (I21)	174 (61.7%)	170 (60.3%)	0.029
Acute kidney failure (N17)	256 (90.8%)	242 (85.8%)	**0.155**
Systolic heart failure, unspecified (I50.20)	69 (24.5%)	89 (31.6%)	**0.158**
Ischemic cardiomyopathy (I25.5)	123 (43.6%)	121 (42.9%)	0.014
Dilated cardiomyopathy (I42.0)	48 (17.0%)	43 (15.2%)	0.048
End-stage heart failure (I50.84)	39 (13.8%)	48 (17.0%)	0.088
Acute pulmonary edema (J81.0)	47 (16.7%)	51 (18.1%)	0.037
Systolic heart failure (I50.2)	205 (72.7%)	195 (69.1%)	0.078
*Respiratory support and resuscitation*			
CPAP < 24 h (5A09357)	19 (6.7%)	17 (6.0%)	0.029
CPAP 24–96 h (5A09457)	≤ 10	≤ 10	< 0.001
CPAP > 96 h (5A09557)	0 (0.0%)	≤ 10	**0.271**
Mechanical ventilation > 96 h (5A1955Z)	114 (40.4%)	129 (45.7%)	**0.108**
Mechanical ventilation 24–96 h (5A1945Z)	54 (19.1%)	49 (17.4%)	0.046
Mechanical ventilation < 24 h (5A1935Z)	27 (9.6%)	24 (8.5%)	0.037
Emergency endotracheal intubation (CPT 31500)	79 (28.0%)	74 (26.2%)	0.040
Endotracheal airway insertion (0BH17EZ)	91 (32.3%)	94 (33.3%)	0.023
Cardiopulmonary resuscitation (CPT 92950)	44 (15.6%)	45 (16.0%)	0.010

Values are n (%) unless otherwise specified. Covariates were ascertained before the index date. The same matched cohorts were used for the 30-day, 90-day, and 1-year analyses. Standardized mean differences (SMD) < 0.10 indicate adequate balance; SMDs ≥ 0.10 are shown in bold. TriNetX reports counts of 1–10 as 10 to protect patient privacy; these cells are shown as ≤ 10, and SMDs derived from them are unreliable. Characteristics before matching are shown in Supplementary Table S1

Before matching, delayed-ECPELLA patients had a higher recorded burden of comorbidity, including heart failure (92.1% vs. 48.3%; SMD 1.09), acute kidney failure (87.4% vs. 44.2%; SMD 1.02), ischemic cardiomyopathy (46.6% vs. 19.9%; SMD 0.59), cardiac arrest (45.7% vs. 23.6%; SMD 0.48), and mechanical ventilation > 96 h (53.7% vs. 12.6%; SMD 0.97). Because covariates were ascertained before the index date, and the index date for delayed ECPELLA was Impella insertion, part of this difference reflects diagnoses recorded during the first days of VA-ECMO support; in the AKI-restricted cohorts indexed to VA-ECMO initiation, pre-matching differences were much smaller (heart failure 49.4% vs. 45.4%; mechanical ventilation > 96 h 5.9% vs. 9.9%, delayed vs. early ECPELLA). After matching, most SMDs were < 0.10; residual imbalance persisted for acute kidney failure (90.8% vs. 85.8%; SMD 0.155), unspecified systolic heart failure (24.5% vs. 31.6%; SMD 0.158), coronary artery disease (75.2% vs. 70.2%; SMD 0.112), mechanical ventilation > 96 h (40.4% vs. 45.7%; SMD 0.108), and heart failure (93.3% vs. 90.4%; SMD 0.104) (early vs. delayed ECPELLA).

### Primary outcome: 30-day mortality

Thirty-day all-cause mortality in the matched cohorts was 48.9% (136 of 278 at risk) in the early-ECPELLA group and 49.6% (139 of 280 at risk) in the delayed-ECPELLA group (risk difference − 0.007, 95% CI − 0.090 to 0.076; RR 0.99, 95% CI 0.83–1.17; *p* = 0.865; Table 2). Thirty-day survival probabilities were 50.3% and 50.1%, respectively (hazard ratio [HR] 0.94, 95% CI 0.74–1.19; log-rank *p* = 0.623; Table 3). Because the proportional-hazards assumption was violated (*p* = 0.018), the RR is the primary estimate.

**Table 2:** Outcomes in the matched cohorts at 30 days, 90 days and 1 year

Outcome	Early ECPELLA, *n*/*N* at risk (%)	Delayed ECPELLA, *n*/*N* at risk (%)	Risk difference (95% CI)	Risk ratio (95% CI)	*p* value
*30-day analysis*					
All-cause mortality	136/278 (48.9)	139/280 (49.6)	–0.007 (–0.090 to 0.076)	0.99 (0.83–1.17)	0.865
Renal-replacement therapy	101/270 (37.4)	49/226 (21.7)	0.157 (0.078 to 0.236)	1.73 (1.29–2.31)	**< 0.001**
Major bleeding	25/226 (11.1)	15/230 (6.5)	0.045 (–0.006 to 0.097)	1.70 (0.92–3.13)	0.087
Durable LVAD implantation	18/282 (6.4)	29/282 (10.3)	–0.039 (–0.085 to 0.006)	0.62 (0.35–1.09)	0.094
Sepsis	20/207 (9.7)	22/198 (11.1)	–0.014 (–0.074 to 0.045)	0.87 (0.49–1.54)	0.633
Limb ischemia	24/214 (11.2)	23/209 (11.0)	0.002 (–0.058 to 0.062)	1.02 (0.59–1.75)	0.945
*90-day analysis*					
All-cause mortality	153/278 (55.0)	153/280 (54.6)	0.004 (–0.079 to 0.087)	1.01 (0.87–1.17)	0.926
Renal-replacement therapy	105/270 (38.9)	53/226 (23.5)	0.154 (0.074 to 0.235)	1.66 (1.26–2.19)	**< 0.001**
Major bleeding	28/226 (12.4)	18/230 (7.8)	0.046 (–0.010 to 0.101)	1.58 (0.90–2.78)	0.106
Durable LVAD implantation	20/282 (7.1)	36/282 (12.8)	–0.057 (–0.106 to − 0.008)	0.56 (0.33–0.94)	**0.024**
Sepsis	23/207 (11.1)	28/198 (14.1)	–0.030 (–0.095 to 0.034)	0.79 (0.47–1.32)	0.358
Limb ischemia	27/214 (12.6)	25/209 (12.0)	0.007 (–0.056 to 0.069)	1.06 (0.63–1.76)	0.837
*1-year analysis*					
All-cause mortality	163/278 (58.6)	160/280 (57.1)	0.015 (–0.067 to 0.097)	1.03 (0.89–1.18)	0.721
Renal-replacement therapy	106/270 (39.3)	54/226 (23.9)	0.154 (0.073 to 0.234)	1.64 (1.25–2.17)	**< 0.001**
Major bleeding	33/226 (14.6)	23/230 (10.0)	0.046 (–0.014 to 0.106)	1.46 (0.89–2.41)	0.134
Durable LVAD implantation	21/282 (7.4)	37/282 (13.1)	–0.057 (–0.107 to − 0.007)	0.57 (0.34–0.95)	**0.027**
Sepsis	25/207 (12.1)	30/198 (15.2)	–0.031 (–0.098 to 0.036)	0.80 (0.49–1.31)	0.367
Limb ischemia	27/214 (12.6)	26/209 (12.4)	0.002 (–0.061 to 0.065)	1.01 (0.61–1.68)	0.956

Patients with the outcome before the relevant follow-up window were excluded from that outcome-specific analysis, accounting for the differing denominators across rows. p values < 0.05 are shown in bold. Limb ischemia was defined as ICD-10-CM I74.3 or I99.8. CI, confidence interval; LVAD, left ventricular assist device

**Table 3:** Kaplan–Meier survival analyses in the matched cohorts

Outcome		Event-free probability, early ECPELLA	Event-free probability, delayed ECPELLA	Hazard ratio (95% CI)			Log-rank *p*		Proportional-hazards *p*
*30-day analysis*									
All-cause mortality		50.3%	50.1%	0.94 (0.74–1.19)*			0.623		0.018
Renal-replacement therapy		61.2%	76.2%	1.92 (1.37–2.70)*			< 0.001		0.029
Major bleeding		87.7%	92.6%	1.70 (0.90–3.23)			0.099		0.970
Durable LVAD implantation		92.8%	87.2%	0.59 (0.33–1.06)			0.071		0.078
Sepsis		88.8%	86.4%	0.84 (0.46–1.54)			0.568		0.416
Limb ischemia		88.0%	87.8%	0.98 (0.55–1.73)			0.948		0.644
*90-day analysis*									
All-cause mortality		43.8%	44.8%	0.97 (0.77–1.21)*			0.769		0.025
Renal-replacement therapy		59.2%	73.7%	1.85 (1.33–2.58)*			**<** 0.001		0.023
Major bleeding		85.6%	90.4%	1.60 (0.88–2.89)			0.117		0.540
Durable LVAD implantation		91.7%	83.1%	0.52 (0.30–0.89)			0.016		0.051
Sepsis		86.1%	81.1%	0.76 (0.44–1.32)			0.321		0.462
Limb ischemia		85.7%	86.2%	1.02 (0.59–1.75)			0.949		0.468
*1-year analysis*									
All-cause mortality		39.6%	41.9%	0.99 (0.79–1.23)*			0.920		0.026
Renal-replacement therapy		58.5%	72.8%	1.84 (1.33–2.56)*			**< **0.001		0.023
Major bleeding		80.6%	85.4%	1.49 (0.88–2.54)			0.138		0.367
Durable LVAD implantation		91.0%	82.1%	0.53 (0.31–0.90)			0.017		0.093
Sepsis		83.7%	78.9%	0.77 (0.45–1.31)			0.330		0.633
Limb ischemia		85.7%	85.3%	0.98 (0.57–1.68)			0.945		0.703

Event-free probability denotes the probability of remaining free from the specified event at the end of the corresponding window. The proportional-hazards assumption was assessed for all analyses. *Assumption violated (*p* < 0.05), for which the risk ratio in Table 2 is the primary estimate. No major violations were identified. CI, confidence interval; LVAD, left ventricular assist device

### Mortality at 90 days and 1 year

At 90 days, mortality was 55.0% with early ECPELLA versus 54.6% with delayed ECPELLA (RR 1.01, 95% CI 0.87–1.17; *p* = 0.926; HR 0.97, 95% CI 0.77–1.21; log-rank *p* = 0.769). At 1 year, mortality was 58.6% versus 57.1% (RR 1.03, 95% CI 0.89–1.18; *p* = 0.721; HR 0.99, 95% CI 0.79–1.23; log-rank *p* = 0.920). One-year survival probabilities were 39.6% in the early-ECPELLA group and 41.9% in the delayed-ECPELLA group (Table 3). The proportional-hazards assumption was violated at both time points (*p* = 0.025 and *p* = 0.026).

### Renal-replacement therapy

In the primary analysis, renal-replacement therapy occurred more frequently in the early-ECPELLA group at every time point. 37.4% versus 21.7% at 30 days (RR 1.73, 95% CI 1.29–2.31; *p* < 0.001), 38.9% versus 23.5% at 90 days (RR 1.66, 95% CI 1.26–2.19; *p* < 0.001), and 39.3% versus 23.9% at 1 year (RR 1.64, 95% CI 1.25–2.17; *p* < 0.001; Tables 2 and 3). Renal-replacement therapy recorded before the index date excluded 56 delayed-ECPELLA patients and 12 early-ECPELLA patients from this analysis. This association was not present in sensitivity analyses in which follow-up began at VA-ECMO initiation in both groups (Section “Sensitivity analysis”).

### Major bleeding

Major bleeding occurred in 11.1% of early-ECPELLA patients and 6.5% of delayed-ECPELLA patients at 30 days (RR 1.70, 95% CI 0.92–3.13; *p* = 0.087), 12.4% versus 7.8% at 90 days (RR 1.58, 95% CI 0.90–2.78; *p* = 0.106), and 14.6% versus 10.0% at 1 year (RR 1.46, 95% CI 0.89–2.41; *p* = 0.134).

### Other outcomes

**Fig. 2:**
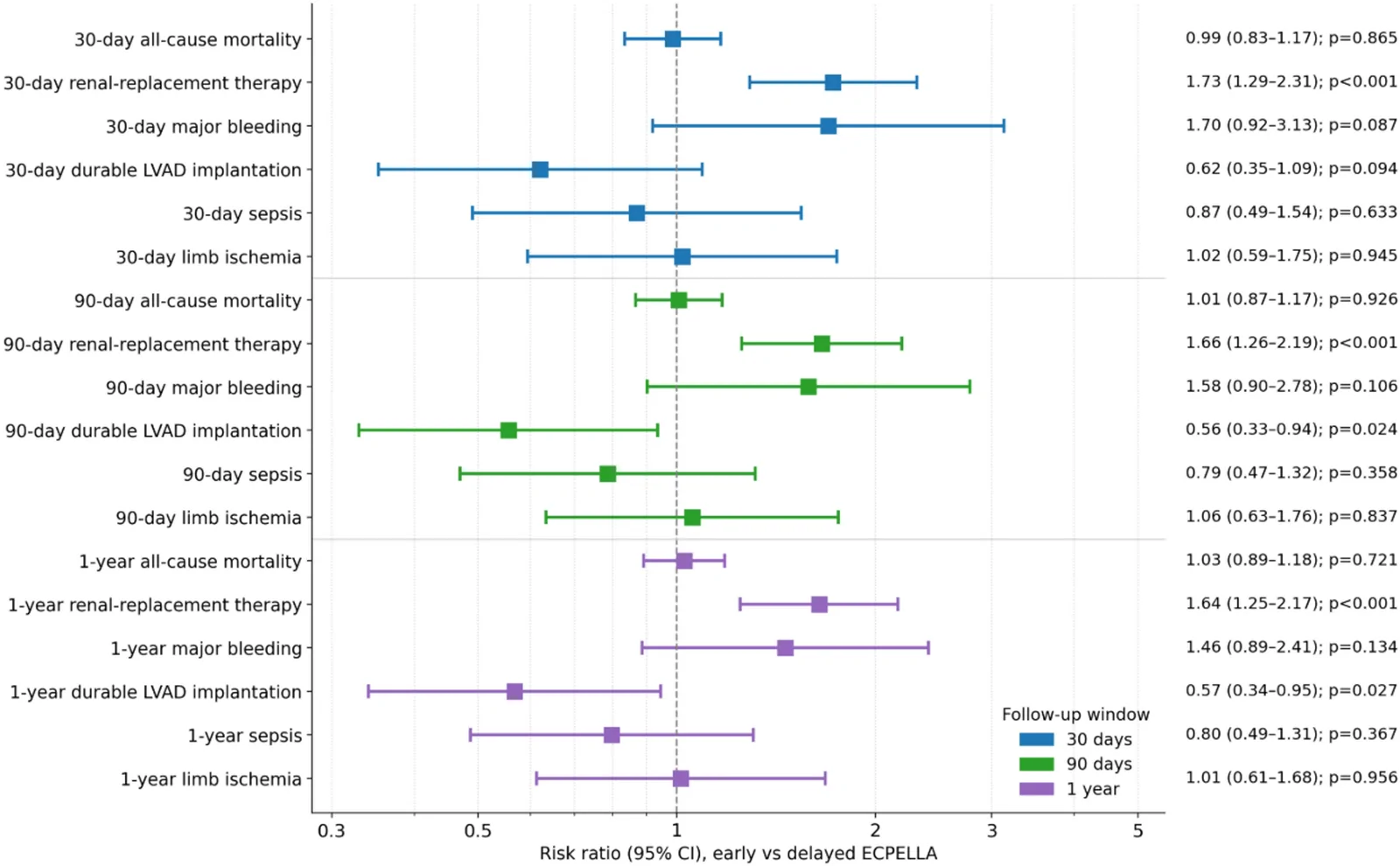
Outcomes of early versus delayed ECPELLA in matched cohorts. Forest plot of risk ratios with 95% confidence intervals for each pre-specified outcome at 30 days, 90 days and 1 year in the matched cohorts (282 per group. Squares denote point estimates and horizontal lines denote 95% confidence intervals. Risk ratio < 1 indicates a lower risk with early ECPELLA; risk ratio > 1 indicates a higher risk with early ECPELLA. RRT, renal-replacement therapy; LVAD, left ventricular assist device

Durable LVAD implantation was less frequent with early ECPELLA at 90 days (7.1% vs. 12.8%; RR 0.56, 95% CI 0.33–0.94; *p* = 0.024) and 1 year (7.4% vs. 13.1%; RR 0.57, 95% CI 0.34–0.95; *p* = 0.027), but not at 30 days (6.4% vs. 10.3%; RR 0.62, 95% CI 0.35–1.09; *p* = 0.094). Sepsis rates were similar between groups across all windows (1-year RR 0.80, 95% CI 0.49–1.31). Limb ischemia was similar between groups at 30 days (11.2% vs. 11.0%), 90 days (12.6% vs. 12.0%), and 1 year (12.6% vs. 12.4%). In the unmatched cohorts, early ECPELLA was associated with more major bleeding, sepsis, and limb ischemia at 30 days; these associations were absent after matching (Supplementary Table S2).The full set of risk ratios with 95% confidence intervals is summarized in Fig. 2.

### Sensitivity analysis

In AKI-restricted cohorts analyzed with the primary index definition (218 per group after matching), early ECPELLA remained associated with more 30-day renal-replacement therapy (43.2% vs. 26.7%; RR 1.62, 95% CI 1.20–2.18). When the index date was set to VA-ECMO initiation in both groups (265 per group after matching), 30-day renal-replacement therapy was similar (41.0% vs. 41.2%; RR 1.00, 95% CI 0.81–1.22), as were mortality (51.0% vs. 48.1%; RR 1.06, 95% CI 0.89–1.26) and durable LVAD implantation (8.3% vs. 8.3%; RR 1.00, 95% CI 0.57–1.76). In the multivariable Cox model with the same index date, early versus delayed ECPELLA was not associated with 30-day renal-replacement therapy (HR 1.02, 95% CI 0.82–1.26; *p* = 0.877), whereas AKI documented before the index date was (HR 1.40, 95% CI 1.08–1.81; *p* = 0.010). In the 7-day landmark analysis (241 per group after matching), mortality from day 8 through day 90 was 42.6% with early ECPELLA and 36.1% with delayed ECPELLA (RR 1.18, 95% CI 0.93–1.50; *p* = 0.175; HR 1.25, 95% CI 0.92–1.70; log-rank *p* = 0.151); renal-replacement therapy (13.8% vs. 14.0%; RR 0.99, 95% CI 0.57–1.70) and durable LVAD implantation (8.1% vs. 11.2%; RR 0.73, 95% CI 0.41–1.27) did not differ (Supplementary Table S3).

## Discussion

In this propensity-matched analysis, early Impella unloading around the time of VA-ECMO initiation was not associated with lower all-cause mortality than Impella placement on days 2–7. Mortality was similar at 30 days, 90 days, and 1 year, and this finding was consistent across sensitivity analyses. Early ECPELLA was associated with more renal-replacement therapy in the primary analysis, but this association disappeared when follow-up in both groups began at VA-ECMO initiation. Major bleeding, sepsis, and limb ischemia did not differ after matching. Durable LVAD implantation was less frequent with early ECPELLA at 90 days and 1 year, but this finding was not reproduced in sensitivity analyses. The high mortality rates, approaching or exceeding 50% in each window, highlight that even with combined mechanical support, outcomes in ECMO-eligible cardiogenic shock remain modest. Future improvements in survival are likely to depend more on system-level care and patient selection than on device timing.

Several lines of evidence have indicated that LV unloading during VA- ECMO can enhance survival. Schrage et al., studying 686 patients with cardiogenic shock across 16 international centers, found that adding Impella to VA-ECMO was linked to lower 30-day mortality, despite increased bleeding and limb ischemia [12]. A follow- up analysis focused on timing revealed that this survival benefit was mainly seen in patients who received Impella before or within two hours of cannulation, with no benefit when unloading was delayed [15]. Similarly, Fiorelli et al.’s meta-analysis across five studies supported these findings on survival but also noted more hemolysis and renal support needs in the ECPELLA group [13]. Russo et al.’s pooled analysis reported comparable results for various unloading strategies [20]. The recent JACC study by Grandin et al., analyzing over 13,000 VA-ECMO cases in the US, observed overall lower mortality with mechanical unloading but no significant difference between early and delayed strategies, though delayed unloading was associated with higher renal injury [14]. Pappalardo et al. also reported a survival benefit in a small, single-center case-matched study, but the group characteristics varied significantly [21].

Our results are neither completely consistent nor entirely contradictory with previous findings. They concur with Grandin et al. in showing no survival advantage for early versus delayed unloading and with Schrage et al. may stem from varied definitions of early unloading (within 2 h of cannulation in that registry versus a calendar-day window in our study), differences in patient populations and shock causes, and unmeasured patient selection factors. It’s also possible that high-volume centers observing survival benefits may not reflect outcomes in broader real-world settings like TriNetX, where Impella decision-making and operator experience are diverse. The variety of unloading methods, including left-atrial cannulation, intra-aortic balloon pump, surgical venting, or transseptal decompression, complicates pooled analyses and underscores the need for evidence tailored to specific devices and timing.

Randomized evidence remains limited and does not establish a survival benefit from routine early unloading during VA-ECMO. EARLY-UNLOAD, which used transseptal left-atrial cannulation rather than Impella, did not reduce 30-day mortality compared with a conventional rescue-unloading strategy [16]. Similarly, ECLS-SHOCK showed that routine early VA-ECMO in infarct-related cardiogenic shock did not improve mortality and increased bleeding and vascular complications, with neutral findings at 1 year [1, 10]. By contrast, DanGer Shock demonstrated a survival benefit with Impella CP in a highly selected population with STEMI-related cardiogenic shock, although adverse events, including renal replacement therapy, were more frequent [2, 11]. Together, these trials emphasize that device type, clinical phenotype and patient selection are likely to be at least as important as timing.

The biological rationale for LV unloading remains compelling. Peripheral VA-ECMO increases aortic pressure and LV afterload, which may worsen LV distension, pulmonary congestion, myocardial wall stress and intracardiac stasis [9]. However, the benefit of adding Impella is unlikely to be uniform. Patients with absent or intermittent aortic-valve opening, progressive pulmonary edema, elevated left-sided filling pressures or failure to wean from VA-ECMO may derive greater hemodynamic benefit than patients with preserved LV ejection or predominant right-ventricular failure [22]. Adding a second large-bore arterial device also introduces competing hazards, including bleeding, vascular injury, hemolysis, and limb ischemia [23]. Other unloading strategies, including intra-aortic balloon counterpulsation, surgical venting and left-atrial decompression, provide different degrees of unloading and have different complication profiles [22, 24]. These distinctions limit extrapolation across studies and support device-specific, phenotype-specific comparisons.

The renal-replacement and bleeding findings require particular caution. Higher rates of hemolysis and renal-replacement therapy have been repeatedly reported with ECPELLA compared with VA-ECMO alone [12, 13]. In our study, the renal-replacement association was not robust. Restricting both cohorts to patients with AKI did not remove it, but it disappeared when follow-up in both groups began at VA-ECMO initiation, in both a matched analysis and a multivariable Cox model. In the primary analysis, follow-up for delayed ECPELLA began at Impella insertion, so renal-replacement therapy started between VA-ECMO initiation and Impella insertion was classified as a pre-existing event, and those patients were excluded from the renal analysis. More delayed-ECPELLA than early-ECPELLA patients were excluded on this basis (56 vs. 12). The primary renal association therefore reflects when follow-up began rather than an effect of early unloading. Major bleeding was more frequent with early ECPELLA before matching but not after, consistent with confounding by baseline severity. These results should not be interpreted as evidence that early unloading causes renal failure or bleeding. Rather, they identify safety outcomes that should be prospectively measured with granular data on hemolysis, anticoagulation, access technique, and device duration.

The comparison is also vulnerable to immortal-time and time-dependent treatment bias. Patients classified as delayed ECPELLA had to remain alive and supported long enough to receive Impella on days 2–7, whereas early-group deaths could occur immediately after VA-ECMO initiation. In the primary analysis, follow-up for delayed ECPELLA also began at Impella insertion, so events between VA-ECMO initiation and Impella insertion were not attributed to that group. Propensity-score matching of baseline variables cannot remove this structural difference. Two sensitivity analyses addressed it. Setting the index date to VA-ECMO initiation in both groups aligned the start of follow-up; mortality remained similar and the renal-replacement difference disappeared. The 7-day landmark analysis restricted the comparison to patients alive 7 days after VA-ECMO initiation and showed no significant mortality difference, but it conditions on survival to the landmark, excludes early deaths and complications, and, because the index date for delayed ECPELLA was Impella insertion, its outcome window began later after VA-ECMO initiation in that group. Neither analysis estimates the effect of early versus delayed unloading from the time of VA-ECMO initiation. The lower rate of durable LVAD implantation with early ECPELLA is susceptible to the same bias, because patients who receive delayed Impella are early survivors and therefore more likely to be evaluated for durable support; this association was not statistically significant in either sensitivity analysis. The findings are best viewed as a comparison of observed clinical pathways rather than a causal estimate of immediate versus delayed unloading. Time-dependent Cox models and target-trial emulation require patient-level exposure timing, which the aggregated TriNetX platform does not provide. Future studies should use time-varying exposure models, marginal structural methods, or target-trial emulation with a clone-censor-weight design, ideally with exact device timestamps and repeated hemodynamic measurements.

Clinically, these findings do not argue against ECPELLA in patients with objective evidence of LV overload. Rather, they caution against assuming that routine Impella placement within the first day improves survival in all patients receiving VA-ECMO. ELSO guidance supports active surveillance for LV distension and selective decompression based on clinical, echocardiographic, and hemodynamic findings [25]. When unloading is required, standardized shock-team pathways should incorporate vascular-access planning, anticoagulation, hemolysis monitoring, distal-perfusion assessment, and early evaluation for myocardial recovery, durable LVAD implantation, or transplantation. Structured cardiogenic-shock protocols and VA-ECMO care bundles have been associated with improved outcomes, although the supporting evidence remains observational [26, 27]. Thus, unloading decisions should be integrated into a broader strategy of patient selection and protocolized care rather than treated as an isolated timing decision.

This study has several limitations. It is retrospective and relies on diagnosis and procedure codes, which may be incomplete or misclassified. TriNetX does not provide the granular information needed to confirm echocardiographic data or SCAI shock stage, AKI severity, creatinine trajectory, or urine output. Although 35 covariates were included in matching, residual confounding remained likely, particularly for acute kidney failure and time-varying shock severity. Because TriNetX records procedure dates without time stamps, exposure windows were defined in calendar days. The early window may combine clinically distinct strategies, including Impella placement before VA-ECMO, simultaneous placement and rescue unloading on the following day and misclassification within both windows is possible. The cohort was not stratified by shock etiology, ECPR status, or post-arrest neurological condition. Outcome definitions were code-based. The limb ischemia definition included a nonspecific circulatory code (I99.8). Some event counts were small, and secondary analyses were not adjusted for multiplicity. Sensitivity analyses were performed after a network update and therefore included slightly different patient populations. Finally, the delayed-exposure definition and primary index created immortal-time bias that could not be fully eliminated. These limitations preclude causal conclusions and reinforce the need for prospective studies with precise device timing and physiological phenotyping.

## Conclusion

In this propensity-matched analysis of a large federated electronic health-record network, immediate Impella unloading at the time of VA-ECMO initiation was not associated with improved survival as compared with delayed unloading. The higher rate of renal-replacement therapy with early ECPELLA in the primary analysis was not present when follow-up in both groups began at VA-ECMO initiation. Residual confounding and immortal-time bias preclude causal interpretation and do not support routine immediate unloading in unselected patients.

## Supplementary Information

Below is the link to the electronic supplementary material.

Supplementary Material 1

## Data Availability

The data supporting the findings of this study were obtained through the TriNetX Global Collaborative Network. The authors did not receive or retain identifiable patient-level data. Access to the underlying TriNetX data is subject to institutional agreements and is not publicly available. Aggregate results generated for this analysis are reported in the manuscript and its accompanying tables and figures.

## Code availability

No custom software code was developed for this study. Cohort construction, propensity-score matching, and outcome analyses were performed using the analytical tools available within the TriNetX platform.
