# Supplementary material for: Early versus delayed impella unloading during venoarterial ECMO for cardiogenic shock: a propensity-matched analysis
Source: J Artif Organs. 2026 Nov 13;29(4):77. doi: 10.1007/s10047-026-01606-2 (PMC13768363; doi:10.1007/s10047-026-01606-2)
Supplement: Supplementary file 1 [file 10047_2026_1606_MOESM1_ESM.docx]

**Supplementary Material**

**Supplementary Table S1. Baseline characteristics before and after 1:1 propensity-score matching**

|  | **Before matching** | | | **After matching** | | |
| --- | --- | --- | --- | --- | --- | --- |
| **Characteristic** | **Early ECPELLA (n = 1,115)** | **Delayed ECPELLA (n = 341)** | **SMD** | **Early ECPELLA (n = 282)** | **Delayed ECPELLA (n = 282)** | **SMD** |
| **Demographics** | | | | | | |
| Age at index, years, mean ± SD | 57.5 ± 13.5 | 57.3 ± 13.4 | 0.016 | 57.7 ± 13.1 | 57.3 ± 13.3 | 0.031 |
| Female | 321 (28.8%) | 90 (26.4%) | 0.054 | 79 (28.0%) | 76 (27.0%) | 0.024 |
| Black or African American | 133 (11.9%) | 33 (9.7%) | 0.073 | 36 (12.8%) | 32 (11.3%) | 0.044 |
| Male | 794 (71.2%) | 251 (73.6%) | 0.054 | 203 (72.0%) | 206 (73.0%) | 0.024 |
| White | 760 (68.2%) | 243 (71.3%) | 0.067 | 196 (69.5%) | 194 (68.8%) | 0.015 |
| American Indian or Alaska Native | ≤10 | ≤10 | 0.149 | ≤10 | ≤10 | <0.001 |
| Unknown race | 101 (9.1%) | 35 (10.3%) | 0.041 | 28 (9.9%) | 29 (10.3%) | 0.012 |
| Native Hawaiian or Other Pacific Islander | 13 (1.2%) | ≤10 | 0.125 | ≤10 | ≤10 | <0.001 |
| Unknown ethnicity | 157 (14.1%) | 33 (9.7%) | 0.136 | 26 (9.2%) | 30 (10.6%) | 0.047 |
| Hispanic or Latino | 69 (6.2%) | 21 (6.2%) | 0.001 | 15 (5.3%) | 17 (6.0%) | 0.031 |
| Not Hispanic or Latino | 889 (79.7%) | 287 (84.2%) | 0.115 | 241 (85.5%) | 235 (83.3%) | 0.059 |
| Other race | 54 (4.8%) | 17 (5.0%) | 0.007 | 12 (4.3%) | 15 (5.3%) | 0.050 |
| Asian | 49 (4.4%) | ≤10 | 0.078 | ≤10 | ≤10 | <0.001 |
| **Comorbidities and acute diagnoses** | | | | | | |
| Essential hypertension (I10) | 501 (44.9%) | 180 (52.8%) | 0.158 | 160 (56.7%) | 153 (54.3%) | 0.050 |
| Diabetes mellitus (E08–E13) | 361 (32.4%) | 149 (43.7%) | 0.235 | 128 (45.4%) | 122 (43.3%) | 0.043 |
| Chronic kidney disease (N18) | 220 (19.7%) | 124 (36.4%) | 0.377 | 101 (35.8%) | 97 (34.4%) | 0.030 |
| Atherosclerotic coronary artery disease (I25.10) | 491 (44.0%) | 248 (72.7%) | 0.608 | 212 (75.2%) | 198 (70.2%) | **0.112** |
| Heart failure, any (I50) | 538 (48.3%) | 314 (92.1%) | 1.091 | 263 (93.3%) | 255 (90.4%) | **0.104** |
| Overweight and obesity (E66) | 318 (28.5%) | 157 (46.0%) | 0.368 | 132 (46.8%) | 123 (43.6%) | 0.064 |
| Tobacco use (Z72.0) | 92 (8.3%) | 45 (13.2%) | 0.160 | 38 (13.5%) | 36 (12.8%) | 0.021 |
| Cardiac arrest (I46) | 263 (23.6%) | 156 (45.7%) | 0.479 | 127 (45.0%) | 117 (41.5%) | 0.072 |
| Acute myocardial infarction (I21) | 436 (39.1%) | 209 (61.3%) | 0.455 | 174 (61.7%) | 170 (60.3%) | 0.029 |
| Acute kidney failure (N17) | 493 (44.2%) | 298 (87.4%) | 1.022 | 256 (90.8%) | 242 (85.8%) | **0.155** |
| Systolic heart failure, unspecified (I50.20) | 139 (12.5%) | 112 (32.8%) | 0.502 | 69 (24.5%) | 89 (31.6%) | **0.158** |
| Ischemic cardiomyopathy (I25.5) | 222 (19.9%) | 159 (46.6%) | 0.591 | 123 (43.6%) | 121 (42.9%) | 0.014 |
| Dilated cardiomyopathy (I42.0) | 78 (7.0%) | 58 (17.0%) | 0.312 | 48 (17.0%) | 43 (15.2%) | 0.048 |
| End-stage heart failure (I50.84) | 57 (5.1%) | 74 (21.7%) | 0.502 | 39 (13.8%) | 48 (17.0%) | 0.088 |
| Acute pulmonary edema (J81.0) | 93 (8.3%) | 71 (20.8%) | 0.359 | 47 (16.7%) | 51 (18.1%) | 0.037 |
| Systolic heart failure (I50.2) | 373 (33.5%) | 249 (73.0%) | 0.864 | 205 (72.7%) | 195 (69.1%) | 0.078 |
| **Respiratory support and resuscitation** | | | | | | |
| CPAP <24 h (5A09357) | 36 (3.2%) | 24 (7.0%) | 0.173 | 19 (6.7%) | 17 (6.0%) | 0.029 |
| CPAP 24–96 h (5A09457) | ≤10 | ≤10 | 0.149 | ≤10 | ≤10 | <0.001 |
| CPAP >96 h (5A09557) | 0 (0.0%) | ≤10 | 0.246 | 0 (0.0%) | ≤10 | **0.271** |
| Mechanical ventilation >96 h (5A1955Z) | 140 (12.6%) | 183 (53.7%) | 0.971 | 114 (40.4%) | 129 (45.7%) | **0.108** |
| Mechanical ventilation 24–96 h (5A1945Z) | 87 (7.8%) | 59 (17.3%) | 0.290 | 54 (19.1%) | 49 (17.4%) | 0.046 |
| Mechanical ventilation <24 h (5A1935Z) | 67 (6.0%) | 34 (10.0%) | 0.147 | 27 (9.6%) | 24 (8.5%) | 0.037 |
| Emergency endotracheal intubation (CPT 31500) | 153 (13.7%) | 98 (28.7%) | 0.374 | 79 (28.0%) | 74 (26.2%) | 0.040 |
| Endotracheal airway insertion (0BH17EZ) | 151 (13.5%) | 128 (37.5%) | 0.572 | 91 (32.3%) | 94 (33.3%) | 0.023 |
| Cardiopulmonary resuscitation (CPT 92950) | 93 (8.3%) | 58 (17.0%) | 0.263 | 44 (15.6%) | 45 (16.0%) | 0.010 |

Values are n (%) unless otherwise specified. Covariates were ascertained before the index date. Standardized mean differences (SMD) ≥0.10 after matching are shown in bold. TriNetX reports counts of 1–10 as 10 to protect patient privacy; these cells are shown as ≤10, and SMDs derived from them are unreliable.

**Supplementary Table S2. Thirty-day outcomes in the unmatched and matched cohorts**

|  | **Unmatched cohorts** | | | **Matched cohorts** |
| --- | --- | --- | --- | --- |
| **Outcome** | **Early ECPELLA, n/N (%)** | **Delayed ECPELLA, n/N (%)** | **Risk ratio (95% CI)** | **Risk ratio (95% CI)** |
| All-cause mortality | 578/1096 (52.7) | 162/338 (47.9) | 1.10 (0.97–1.25) | 0.99 (0.83–1.17) |
| Renal-replacement therapy | 361/1079 (33.5) | 59/269 (21.9) | **1.53 (1.20–1.94)** | **1.73 (1.29–2.31)** |
| Major bleeding | 200/1005 (19.9) | 18/268 (6.7) | **2.96 (1.87–4.71)** | 1.70 (0.92–3.13) |
| Durable LVAD implantation | 55/1115 (4.9) | 33/340 (9.7) | **0.51 (0.34–0.77)** | 0.62 (0.35–1.09) |
| Sepsis | 164/961 (17.1) | 23/237 (9.7) | **1.76 (1.16–2.66)** | 0.87 (0.49–1.54) |
| Limb ischemia | 194/996 (19.5) | 26/250 (10.4) | **1.87 (1.27–2.75)** | 1.02 (0.59–1.75) |

Unmatched cohorts: 1,115 early and 341 delayed ECPELLA patients; matched cohorts: 282 per group. Patients with the outcome before the follow-up window were excluded from that outcome-specific analysis. Risk ratios with p<0.05 are shown in bold. Limb ischemia was defined as ICD-10-CM I74.3 or I99.8.

**Supplementary Table S3. Sensitivity analyses**

| **Analysis and outcome** | **Early ECPELLA, n/N (%)** | **Delayed ECPELLA, n/N (%)** | **Estimate (95% CI)** | **p value** |
| --- | --- | --- | --- | --- |
| **A. AKI-restricted cohorts, primary index date (816 vs 269 before matching; 218 per group after matching)** | | | | |
| 30-day all-cause mortality | 112/217 (51.6) | 109/217 (50.2) | RR 1.03 (0.85–1.24) | 0.773 |
| 30-day renal-replacement therapy | 89/206 (43.2) | 44/165 (26.7) | RR 1.62 (1.20–2.18) | 0.001 |
| **B. AKI-restricted cohorts, index date = VA-ECMO initiation in both groups (817 vs 269 before matching; 265 per group after matching)** | | | | |
| 30-day all-cause mortality | 134/263 (51.0) | 126/262 (48.1) | RR 1.06 (0.89–1.26) | 0.512 |
| 30-day renal-replacement therapy | 107/261 (41.0) | 107/260 (41.2) | RR 1.00 (0.81–1.22) | 0.971 |
| 30-day durable LVAD implantation | 22/265 (8.3) | 22/265 (8.3) | RR 1.00 (0.57–1.76) | 1.000 |
| **C. Multivariable Cox model, AKI-restricted cohorts, index date = VA-ECMO initiation (817 vs 269), outcome 30-day renal-replacement therapy** | | | | |
| Early vs delayed ECPELLA |  |  | HR 1.02 (0.82–1.26) | 0.877 |
| Acute kidney failure before index |  |  | HR 1.40 (1.08–1.81) | 0.010 |
| Heart failure |  |  | HR 0.81 (0.63–1.05) | 0.117 |
| Male sex |  |  | HR 1.01 (0.82–1.25) | 0.937 |
| **D. 7-day landmark: alive 7 days after VA-ECMO initiation, outcomes day 8–90 (771 vs 290 before matching; 241 per group after matching)** | | | | |
| All-cause mortality | 95/223 (42.6) | 70/194 (36.1) | RR 1.18 (0.93–1.50) | 0.175 |
| Renal-replacement therapy | 22/159 (13.8) | 23/164 (14.0) | RR 0.99 (0.57–1.70) | 0.961 |
| Durable LVAD implantation | 19/234 (8.1) | 26/232 (11.2) | RR 0.73 (0.41–1.27) | 0.259 |
| Sepsis | 15/164 (9.1) | 24/166 (14.5) | RR 0.63 (0.34–1.16) | 0.135 |

All analyses except C used 1:1 propensity-score matching on the covariates listed in Section 2.4. In analysis A, the index date was defined as in the primary analysis; in analyses B and C, follow-up began at VA-ECMO initiation in both groups; in analysis D, outcomes were assessed from day 8 through day 90 after the index date as defined in the primary analysis. In analysis A, AKI in the delayed cohort was required on or before the first date on which cardiogenic shock, VA-ECMO, and AKI were all recorded. Analysis C was fitted in the unmatched AKI-restricted cohorts and adjusted for sex, acute kidney failure, and heart failure recorded before the index date. AKI, acute kidney injury; HR, hazard ratio; LVAD, left ventricular assist device; RR, risk ratio.
